# Role of APD-Ribosylation in Bone Health and Disease

**DOI:** 10.3390/cells8101201

**Published:** 2019-10-05

**Authors:** Chun Wang, Gabriel Mbalaviele

**Affiliations:** Division of Bone and Mineral Diseases, Washington University School of Medicine, St. Louis, MO 63110, USA; chun.wang@wustl.edu

**Keywords:** ARTDs, ADP-ribosylation, bone, osteoclasts, osteoblasts, adipocytes

## Abstract

The transfer of adenosine diphosphate (ADP)-ribose unit(s) from nicotinamide adenine dinucleotide (NAD^+^) to acceptor proteins is known as ADP-ribosylation. This post-translational modification (PTM) unavoidably alters protein functions and signaling networks, thereby impacting cell behaviors and tissue outcomes. As a ubiquitous mechanism, ADP-ribosylation affects multiple tissues, including bones, as abnormal ADP-ribosylation compromises bone development and remodeling. In this review, we describe the effects of ADP-ribosylation in bone development and maintenance, and highlight the underlying mechanisms.

## 1. Introduction

Adenosine diphosphate (ADP)-ribosylation is the transfer of ADP-ribose units from nicotinamide adenine dinucleotide (NAD^+^) to acceptor proteins [1,2]. This post-translational modification (PTM) either occurs as mono-ADP-ribosylation (MARylation) or poly-ADP-ribosylation (PARylation) upon attachment of a single or up to 200 ADP-riboses to targeted proteins, respectively [1,2,3,4,5,6]. ADP-ribosylation is catalyzed by ADP-ribosyltransferases (ARTs), which include diphtheria toxin-like ARTs (ARTDs), also known as poly(ADP-ribose) polymerases (PARPs), and cholera toxin-like ARTs (ARTCs), and some sirtuins (SIRTs) [1,2]. The human genome encodes 17 ARTDs, 4 ARTCs, and 7 SIRT members [1,2,7]. While PARylation, which can be linear or branched, is carried out by ART family members such as ARTD1, ARTD2, ARTD5, and ARTD6, MARylation is the function of a subset of ARTDs (e.g., ARTD3, ARDT10, and ARTD14), ARTCs (ARTC1 and ARTC5), and SIRTs (SIRT4 and SIRT6) [1,2,7,8]. ADP-ribosylation is reversible, as ADP-riboses are removed from MARylated proteins by enzymes such as ADP-ribosyl hydrolases (ARH1 and ARH3), terminal ADP-ribose protein glycohydrolase 1 (TARG1), macrodomains (MacroD1 and MacroD2), and from PARylated proteins by ARH3 and poly(ADP-ribose) glycohydrolase (PARG) [2,8,9]. By modifying or regulating proteins endowed with structural roles (e.g., histones), signaling functions, (e.g., mitogen-activated protein kinases, MAPKs), or transcriptional activities (e.g., transcription factors) [10,11,12,13,14], ARTs profoundly influence the whole organism homeostasis through regulation of countless cellular events, including transcription, replication, proliferation, differentiation, and survival [13,15,16,17]. Structural homologies, activity specificities, and cellular distributions of ARTs, as well as non-skeletal pathologies caused by these proteins, have been comprehensively reviewed elsewhere [1,2,4,16,18,19], therefore, this review does not discuss these topics, but focuses on bone regulation by ADP-ribosylation.

**Figure 1:**
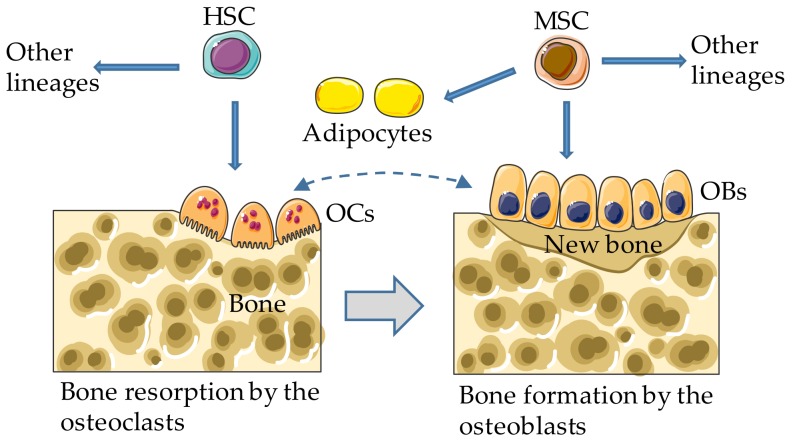
Bone remodeling. In adults, the osteoclasts (OCs) and other hematopoietic lineages (not depicted) arise from bone marrow hematopoietic stem cells (HSCs); the osteoblasts differentiate from mesenchymal stem cells (MSCs), which can also develop into adipocytes and other lineages (not depicted). Dashed and gray arrows indicate bidirectional regulatory interactions and coupling between bone resorption and formation, respectively.

During development, mesenchymal condensations ossify directly or indirectly via a cartilaginous template, embryonic events known as intramembranous and endochondral ossification, respectively, where bone formation by the osteoblasts dominates bone resorption by the osteoclasts [20,21,22]. Postnatally, particularly during adulthood, bone resorption is offset by bone formation, a coupling process that preserves bone mass and biomechanical properties (Figure 1). An imbalance between bone resorption and formation underlies a variety of diseases featured by excessive bone gain or loss [23,24,25]. While the osteoclasts arise from hematopoietic stem cells, the osteoblasts derive from mesenchymal stem cells (MSCs), which can also differentiate into adipocytes and chondrocytes under the influence of specific environmental cues [26,27], as detailed below. Excessive bone marrow adipogenesis at the expenses of osteogenesis has deleterious effects on bone, a tissue in which various cell types, including mesenchymal and hematopoietic cells, express a repertoire of ARTs and SIRTs. We review the impact of ADP-ribosylation on the differentiation of the osteoclasts, osteoblasts, and adipocytes, focusing on ART and SIRT members with a functional link to bone health and disease.

## 2. Osteoclast Differentiation

During development, the emerging ossification centers recruit myeloid progenitors where they undergo terminal differentiation into the osteoclasts, which resorb the mineralized matrix, an action that over time results in the formation of the bone marrow cavity [28,29,30]. Postnatally, the damaged or old bone matrix is sensed and removed by the osteoclasts, and is evenly replaced by the osteoblasts. Potential sensors of defective bone matrix components include the innate immune complex, NOD-like receptor family (NLR), pyrin domain containing 3 (NLRP3) inflammasome, which is activated by bone matrix degradation products and promotes osteoclast differentiation [31,32]. While bone marrow myeloid precursors (e.g., CD11b^low^CD115^high^CD117^high^-expressing cells) differentiate into the osteoclasts in homeostatic conditions, circulating monocytes are capable of forming osteoclasts or fusing with pre-existing multinucleated osteoclasts in pathological settings, such as inflammatory arthritis [33,34,35,36,37,38]. Osteoclast differentiation, activity, and survival depend on macrophage colony-stimulating factor (M-CSF) and receptor activator of NF-κB ligand (RANKL), whose expression and signaling outputs are regulated by various factors such as hormones (e.g., parathyroid hormone, estrogen, and 1,25α-dihydroxyvitamin D3) and pro-inflammatory cytokines, including those of the tumor necrosis factor (TNF) and interleukin-1 (IL-1) families [23,24,25,39]. For simplicity, this review focuses on osteoclast differentiation, though other biological aspects of these cells, such as activity and survival, are occasionally described. M-CSF, RANKL, and the majority of osteoclast-regulating factors are mainly produced by cells of the osteoblast lineage, and immune cells (e.g., macrophages, T and B lymphocytes) though the osteoclasts themselves produce factors such as sphingosine-1-phosphate and Wnts, which act not only in autocrine manner, but also paracrine fashion, regulating the functions of neighboring cells such as the osteoblasts [40,41,42,43,44,45]. Osteoclast differentiation is driven by complex interactions among various transcription factors, including the nuclear factor of activated T cells cytoplasmic 1 (NFATc1), NF-κB, and c-Fos [46]. While the effects of PTMs such as phosphorylation, methylation, ubiquitination, and SUMOylation on transcriptional regulation and other key osteoclastogenic events have been extensively studied [47,48,49,50,51,52,53,54,55], only a few studies have investigated the role of ADP-ribosylation in osteoclast biology.

### 2.1. Role of ARTD1 in Osteoclast Differentiation

ARTD1 is the most studied member of the ARTD family in the skeleton. Early studies show that ARTD1 protein levels decline during in vitro osteoclast differentiation induced by RANKL, a response that correlates with increased expression of the a3 isoform of the V-ATPase subunit, tartrate-resistant acid phosphatase, and brain-type creatine kinase [56,57,58,59]. In agreement with the proposition that ARTD1 is a negative regulator of osteoclastogenesis, this protein binds to and represses the activity of the promoters of the aforementioned genes in the osteoclast precursors [56,57,58,59]. Follow up studies using engineered mice expressing uncleavable ARTD1 or *Artd1*-deficient mice, not only reinforce the anti-osteoclastogenic functions of ARTD1, but also shed light into the underlying mechanisms [60,61,62,63]. Novel insights include the demonstration that i) ARTD1 inhibits histone3lysine4 trimethylation (H3K4me3), histone marks of active chromatin, at the promoters of key osteoclastogenic factors such as B lymphocyte-induced maturation protein 1 (Blimp1), and ii) ARTD1 PARylates itself during osteoclast formation, a prerequisite modification that targets this protein for destruction through the proteasome pathway [63]. ARTD1 also inhibits H3K4me3 and H4 acetylation, thereby impeding the recruitment of the RelA subunit of NF-κB to the IL-1β promoter [61]. Progressive decline in ARTD1 levels also occurs during the differentiation of myotubes, which are multinucleated fibers that arise from the fusion of myoblasts [64]. The basis for the apparent inverse correlation between ARTD1 abundance and multinucleation is unclear, though it is tempting to speculate that the degradation of this enzyme, whose activity can deplete total intracellular NAD^+^ levels by 80% may be necessary to prevent energy collapse during the high energy-demanding differentiation process.

Mice lacking ARTD1 globally or selectively in myeloid cells indistinguishably exhibit a low bone mass phenotype associated with an increased number of the osteoclasts on bone surfaces (Wang et al., personal communication). Consistent with the view of osteoclast lineage autonomous actions of ARTD1, in vitro osteoclastogenesis from isolated mouse bone marrow cells is higher in *Artd1* null cells compared to wild-type controls [61]. Potential ARTD1 substrates include the master regulators of osteoclast differentiation, NF-κB and NFATc1, which are PARylated by this enzyme in T cells and smooth muscle cells [65,66,67,68,69]. However, such interplay is unlikely in light of the recent study indicating that PARylated NF-κB and NFATc1 are undetectable in cells of the osteoclast lineage. Instead, ARTD1 consistently PARylates histone H2B among other proteins, and decreases the occupancy of H2B at the NFATc1 promoter, thereby inhibiting NFATc1 expression and restraining osteoclast differentiation (Wang et al., personal communication).

**Figure 2:**
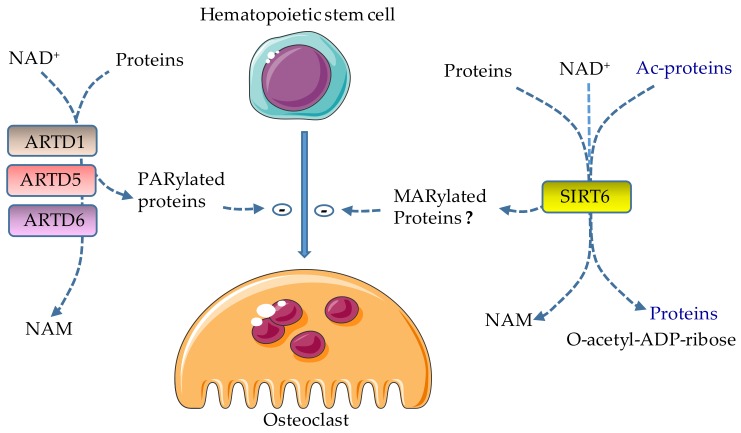
Effects of ADP-ribosylation on osteoclast formation. ARTD1, ARTD5, and ARTD6 catalyze the attachment of ADP-ribose polymers from NAD^+^ to target proteins (PARylation), releasing nicotinamide (NAM) in the process; their actions lead to the inhibition of osteoclast differentiation. SIRT6 inhibits osteoclast development; however, the effects of its MARylating actions in this process are not clear because this enzyme also has deacetylase activity. Lysine deacetylation is coupled to NAD^+^ hydrolysis, yielding a deacetylated targeted protein, O-acetyl-ADP-ribose, and NAM. Ac, acetyl.

ARTD1 is cleaved at D214 into 89 kDa and 24 kDa fragments, presumably by caspase-7, in response to activation of the NLRP3 and NLR, CARD containing 4 (NLRC4) inflammasomes [63,70,71,72]. Consistent with its pro-inflammatory actions, loss of ARTD1 partially protects joints from destruction in the mouse model of collagen antibody-induced arthritis [73,74,75,76,77]. In line with the ability of ARTD1 and its cleaved fragments to activate signaling platforms such as the NF-κB pathway, knockin mice expressing uncleavable ARTD1 are resistant to ischemia/reperfusion-induced inflammation in intestine and kidney [60,65]. Unexpectedly, this ARTD1 mutant does not affect inflammatory outcomes induced by hyperactive NLRP3 inflammasome [62]. These conflicting results may be explained by the fact that ARTD1 actions are cell-context-dependent. Indeed, ARTD1 promotes NF-κB PARylation or activity in cultured smooth muscle cells, neuronal cells, and macrophages, while negatively regulating this transcription factor in lymphocytic leukemia cells [65,66,68,72]. Despite some gaps in our understanding of ARTD1 mechanisms of action, evidence overwhelmingly indicates that this enzyme negatively regulates osteoclast development (Figure 2).

### 2.2. Role of ARTD5 and ARTD6 in Osteoclast Differentiation

ARTD5 (also known as PARP5A or tankyrase 1) and ARTD6 (also referred to as PARP5B or tankyrase 2) [8] are expressed by many cell types, including the osteoclast lineage [78,79,80,81]. ARTD5 and ARTD6 are implicated in a range of biological processes, including DNA repair, glucose homeostasis and energy expenditure, and skeletal metabolism (through their interactions with the adaptor protein SH3 domain-binding protein 2, SH3BP2 and AXIN 1/2) [78,82,83,84,85,86]. PARylation targets SH3BP2 for ubiquitination by the E3-ubiquitin ligase RNF46, and subsequently for degradation [78,87]. Missense mutations in *SH3BP2* result in SH3BP2 that is stable, as it escapes the destructive actions of ARTD5 and ARTD6, and are associated with cherubism, a hereditary childhood-onset autoinflammatory disorder, whose severity regresses after puberty [88]. Focal facial bone lesions and deformities associated with the destruction of the jaws and dental complications characterize this disease [88]. Knockin mice expressing the most common disease-associated allele develop systemic inflammation (e.g., excessive TNF-α production) and bone loss due to massive osteoclast differentiation as a consequence of heightened sensitivity to M-CSF- and RANKL-induced signals; these events ultimately cumulate in hyperactivation of osteoclastogenic pathways such as Src, Syk, ERK1/2, and NFATc1 [78,79]. Conversely, *Sh3bp2-deficient* osteoclasts exhibit defective bone resorption in vitro [80]. Furthermore, pharmacological inhibition of ARTD5 and ARTD6, which results in SH3BP2 accumulation, promotes osteoclast differentiation in vitro and bone resorption in vivo [81,89], findings that are consistent with accelerated in vitro osteoclastogenesis of osteoclast precursors lacking both ARTD5 and ARTD6 [78]. A recent study suggests that oral bacteria produce pathogen-associated molecular patterns (PAMPs), which in conjunction with danger-associated molecular patterns (DAMPs) released during the remodeling of the jaws, provide tissue-restricted bone lesions in cherubism. Decreased jaw remodeling with age leading to attenuated levels of DAMPs may underlie the reported regression of this disorder over time in the affected patients [90]. Thus, ARTD5 and ARTD6 function as negative regulators of osteoclast differentiation (Figure 2).

### 2.3. Role of SIRT6 in Osteoclast Differentiation

SIRTs are involved in the regulation of insulin secretion, gluconeogenesis, transcriptional regulation, and several other biological responses [91,92,93,94,95]. Relevant to this review are SIRT4 and SIRT6, owing to their MARylation activity that targets numerous proteins including glutamate dehydrogenase and ARTD1 [91,96,97,98]. SIRT6 also MARylates itself, a presumed mechanism of self-regulation [99]. Notably, SIRT6, but not SIRT4, has a strong deacetylase activity, a reaction where lysine deacetylation is coupled to NAD^+^ hydrolysis yielding O-acetyl-ADP-ribose, nicotinamide, and a deacetylated targeted protein [100,101,102,103].

Consistent with SIRT6 inhibitory effects on the transactivation of NF-κB, an important regulator of osteoclast development, overexpression of SIRT6 suppresses RANKL-induced OC formation in vitro and bone destruction in mice with collagen-induced arthritis [25,104,105,106]. Conversely, SIRT6 deficiency causes premature aging associated with increased osteoclastogenesis and low bone mass, or osteopenia associated with low bone turnover [107,108,109,110]. Moreover, myeloid-specific deletion of SIRT6 results in a decrease in estrogen receptor α protein levels and apoptosis of pre-osteoclasts, resulting in massive bone resorption during aging and following ovariectomy [111]. Accordingly, SIRT6 transgenic mice are protected from ovariectomy-induced bone loss. Mechanistically, SIRT6 deacetylates estrogen receptor α at K171 and K299, thereby preventing its proteasomal degradation. In contrast to these studies, SIRT6 reportedly forms a complex with Blimp1 to negatively regulate the expression of anti-osteoclastogenic genes such as V-maf musculoaponeurotic fibrosarcoma oncogene homolog B (Mafb), consistent with the increased bone mass and decreased osteoclast number in mice lacking SIRT6 in hematopoietic cells [112]. Conversely, retroviral-mediated overexpression of SIRT6 increases osteoclast formation [112]. Thus, SIRT6 regulates osteoclast differentiation, but the extent to which SIRT6-driven MARylation affects osteoclastogenesis is unclear given the deacetylase activity of this enzyme (Figure 2).

## 3. Osteoblast Differentiation

The osteoblasts differentiate from MSCs when exposed to growth factors such as bone morphogenetic proteins (BMPs), Wnts, Hedgehog, and Notch, which activate transcription factors such as RUNX2, osterix (OSX), and β-catenin [113]. Unlike osteoclastogenesis, osteoblast differentiation and function are regulated negatively by inflammatory signals, some of which are mediated by ARTs [114,115].

### 3.1. Role of ARTD1 in Osteoblast Differentiation

**Figure 3:**
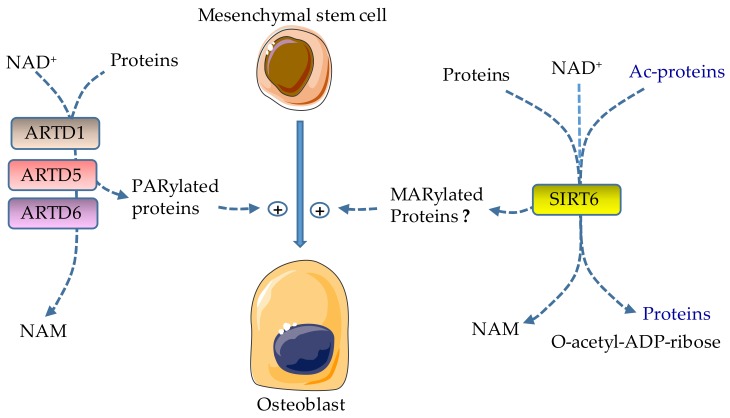
Effects of ADP-ribosylation on osteobast formation. ARTD1, ARTD5, and ARTD6 catalyze the attachment of ADP-ribose polymers from NAD^+^ to target proteins (PARylation), releasing nicotinamide (NAM) in the process; their activities promote osteoblast differentiation. SIRT6 promotes osteoblast development; however the effects of MARylation driven by SIRT6 in this process are not clear because this enzyme also has deacetylase activity. Lysine deacetylation is coupled to NAD^+^ hydrolysis, yielding a deacetylated targeted protein, O-acetyl-ADP-ribose, and NAM. Ac, acetyl.

Immunohistochemical analysis of human bone samples shows that ARTD1 is mostly expressed in osteoblasts in the areas of new bone formation, to a lesser extent in osteoclasts, while no positive staining is detected in osteocytes, suggesting a role for ARTD1 in bone formation [116]. Poly-ADP-ribose (PAR) motifs are unexpectedly detected by nuclear magnetic resonance (NMR) and immunostaining in the bone extracellular matrix, mostly in the calcifying region of the growth plate, but to a lesser degree in the adjoining nonmineralized hypertrophic cartilage [117]. Thus, PAR units, which may be released during cell necrosis, are potentially implicated in bone matrix calcification. Earlier studies using human MSCs and SAOS-2 cells show that during osteoblast differentiation, hydrogen peroxide activates ARTD1 and promotes osteoblastogenesis via activation of the p38 MAPK pathway [118,119,120]. Consistent with its bone anabolic actions, the recruitment of ARTD1 by the long non-encoding RNA (lncRNA) STEEL results in increased angiogenesis and fracture healing [121]. Furthermore, the ARTD inhibitor PJ34 suppresses osteogenic differentiation of murine MSCs, but does not affect chondrocyte or adipocyte differentiation [122]. Thus, ARTD1 promotes osteoblast differentiation under physiological conditions. However, ARTD1 also interacts with NF-κB in mediating TNF-induced suppression of phosphate-regulating gene with homologies to endopeptidases on the X chromosome (Phex), whose important functions in bone mineralization include the inhibition of the expression of the hypophosphatemic fibroblast growth factor 23 (FGF23) [123,124]. Thus, while ARTD1 favors osteogenesis in homeostatic conditions (Figure 3), it may compromise this process in inflammatory states.

### 3.2. Role of ARTD5 and ARTD6 in Osteoblast Differentiation

ARTD5 and ARTD6 PARylate and destabilize AXIN, a negative regulator of the critical osteogenic Wnt/β-catenin signaling pathway [125]. Bone formation is impaired both in vivo and in vitro in *Sh3bp2*-deficient cells through mechanisms involving the tyrosine kinase ABL, and the transcription factors TAZ and RUNX2 [80,126]. The inhibitors of ARTD5 and ARTD6 enhance in vitro osteoblastogenesis as the result of accumulated SH3BP2, promote nuclear translocation of ABL, TAZ, and RUNX2, but they paradoxically decrease bone mass in mice associated with an increased number of osteoclasts [81]. Thus, by stabilizing AXIN and SH3BP2, the inhibitors of ARTD5 and ARTD6 have the potential of inhibiting osteogenesis (Figure 3) and inflicting substantial damage to the skeleton.

### 3.3. Role of SIRT6 in Osteoblast Differentiation

SIRT6-deficient mice exhibit stunted growth as a result of abnormal development of the growth plate and impaired bone formation [108,109,110,127]. This phenotype is consistent with the plethoric actions of this enzyme. Indeed, SIRT6 regulates the expression of RUNX2 and OSX through deacetylation of H3K9; its deficiency is associated with hyperacetylation of H3K9 at the promoter of dickkopf-related protein 1 (Dkk1), a potent negative regulator of osteoblastogenesis [110]. SIRT6 also modulates the expression of the components of BMP signaling, actions that are p300/CBP-associated factor (PCAF)-dependent [128]. Finally, SIRT6 promotes osteogenic differentiation of rat bone marrow MSCs partially via suppression of NF-κB [129]. Thus, the actions of SIRT6 are pro-osteogenic osteoblastogenesis. However, given the importance of protein deacetylation in osteogenesis, a function that is also carried out by SIRT6, the role of MARylation mediated by this enzyme in this process is unclear.

## 4. Adipocyte Differentiation

Bone marrow adiposity and visceral fat are implicated in the pathogenesis of bone diseases such as osteoporosis [130,131]. Excessive differentiation of MSCs towards adipocytes in conjunction with the secretion of adipokines (e.g., adiponectin) and cytokines (e.g., IL-6) adversely impact bone metabolism. The adipogenic differentiation program of MSCs is controlled by transcription factors such as CCAAT/enhancer binding protein α (C/EBPα) and peroxisome proliferator-activated receptor γ (PPARγ) [132,133].

### Role of ARTD1 in Adipocyte Differentiation

ARTD1 activity drops for several hours during the early phase of preadipocytes 3T3-L1 cell differentiation into adipocytes before returning to baseline levels and subsequently reaching higher levels, presumably as a result of chromatin modifications [134]. Studies using stromal cells from the fat pads of ARTD1-deficient mice show that the loss of this enzyme is associated with impaired adipocyte function and differentiation [135]. Accordingly, when fed with a high-fat diet, *Artd1* mice develop hepatosteatosis and dysregulated glucose metabolism. Using the preadipocyte 3T3-L1 cells, this group further demonstrates that ARTD1 is recruited to the promoters of *PPARγ2* and its target genes such as *CD36* and *aP2* in a PAR-dependent manner, responses that correlate with decreased histone marks of repressed chromatin (H3K9me3), while marks of active chromatin (H3K4me3) are increased [136]. However, studies based on a different mouse line suggest that lack of ARTD1 increases energy expenditure through SIRT1 activation [137], findings that are consistent with the browning of primary white adipocytes in vitro by olaparib, an inhibitor of ARTD1 and ARTD2 [138]. Other studies also show that ARTD1 PARylates C/EBPβ, thereby inhibiting its DNA binding and transcriptional activities, and ultimately, adipogenesis [139]. Thus, ARTD1 plays various roles in the differentiation of adipocytes, acting at different stages to promote or inhibit this process.

## 5. Therapeutic Implications

ARTs are novel and promising targets for cancer therapies. Numerous studies have shown that various small-molecule inhibitors of ARTDs are efficacious against various cancers, including ovarian cancer, breast cancer, colon cancer, lung cancer, prostate cancer, hepatocellular carcinoma, osteosarcoma, and chordoma [140,141,142,143,144,145,146,147,148,149,150,151]. More importantly, the US Food and Drug Administration has approved three different ARTD inhibitors, olaparib, niraparib, and rucaparib for the treatment of BRCA1- or BRAC2-mutated ovarian cancers. Considering the crucial role that ARTDs play in the pathogenesis of acute tissue injury or periodontitis, some of these drugs may be indicated for the treatment of inflammatory osteolysis. However, pre-clinical evidence indicates that genetic or pharmacological inhibition of ARTD1 or ARTD5 and ARTD6 causes bone loss, a high-risk factor for fracture. Therefore, comprehensive translational studies may help understand the extent to which ARTD inhibitors may adversely affect the skeleton.

## Abbreviations

ADP: adenosine diphosphate; ARH, ADP-ribosyl hydrolase; ART, ADP-ribosyltransferase; ARTD, diphtheria toxin-like ART; ARTC, cholera toxin-like ART; Blimp1, B lymphocyte induced maturation protein 1; BMPs, bone morphogenetic proteins; C/EBPα, CCAAT/enhancer binding protein α; DAMPs, danger-associated molecular patterns; Dkk1, dickkopf-related protein 1; IL-1, interleukin-1; lncRNA, long non-encoding RNA; Mafb, V-maf musculoaponeurotic fibrosarcoma oncogene homolog B; MAPK, mitogen-activated protein kinase; MARylation, mono-ADP-ribosylation; M-CSF, macrophage colony-stimulating factor; MSC, mesenchymal stem cell; NFATc1, nuclear factor of activated T cells cytoplasmic 1; PAMPs, pathogen-associated molecular patterns; NAM, nicotinamide; NLRC4, NLR, CARD containing 4; NLRP3, NOD-like receptor family (NLR), pyrin domain containing 3; PARG, poly(ADP-ribose) glycohydrolase; OSX, osterix; PAR, poly-ADP-ribose; PARP, poly(ADP-ribose) polymerase; PARylation, poly-ADP-ribosylation; PCAF, p300/CBP-associated factor; Phex, phosphate-regulating gene with homologies to endopeptidases on the X; PM, post-translational modification; PPARγ, peroxisome proliferator-activated receptor γ; RANKL, receptor activator of NF-κB ligand; SH3BP2, SH3 domain-binding protein 2; SIRT, sirtuin; TARG1, terminal ADP-ribose protein glycohydrolase 1; TNF, tumor necrosis factor.

## References

[1] Kunze F.A., Hottiger M.O. (2019). Regulating Immunity via ADP-Ribosylation: Therapeutic Implications and Beyond. Trends Immunol..

[2] Bütepage M., Eckei L., Verheugd P., Lüscher B. (2015). Intracellular Mono-ADP-Ribosylation in Signaling and Disease. Cells.

[3] Cohen M.S., Chang P. (2018). Insights into the biogenesis, function, and regulation of ADP-ribosylation. Nat. Chem. Biol..

[4] Gupte R., Liu Z., Kraus W.L. (2017). PARPs and ADP-ribosylation: Recent advances linking molecular functions to biological outcomes. Genes Dev..

[5] Vivelo C.A., Leung A.K. (2015). Proteomics approaches to identify mono-(ADP-ribosyl)ated and poly(ADP-ribosyl)ated proteins. Proteomics.

[6] Alemasova E.E., Lavrik O.I. (2019). Poly(ADP-ribosyl)ation by PARP1: Reaction mechanism and regulatory proteins. Nucleic Acids Res..

[7] Di Girolamo M., Fabrizio G. (2019). Overview of the mammalian ADP-ribosyl-transferases clostridia toxin-like (ARTCs) family. Biochem. Pharmacol..

[8] Hottiger M.O. (2015). Nuclear ADP-Ribosylation and Its Role in Chromatin Plasticity, Cell Differentiation, and Epigenetics. Annu. Rev. Biochem..

[9] Crawford K., Bonfiglio J.J., Mikoč A., Matic I., Ahel I. (2018). Specificity of reversible ADP-ribosylation and regulation of cellular processes. Crit. Rev. Biochem. Mol. Biol..

[10] Bartlett E., Bonfiglio J.J., Prokhorova E., Colby T., Zobel F., Ahel I., Matic I. (2018). Interplay of Histone Marks with Serine ADP-Ribosylation. Cell Rep..

[11] Abplanalp J., Hottiger M.O. (2017). Cell fate regulation by chromatin ADP-ribosylation. Semin. Cell Dev. Boil..

[12] Racz B., Hantó K., Tapodi A., Solti I., Kálmán N., Jakus P., Kovacs K., Debreceni B., Gallyas F., Sumegi B. (2010). Regulation of MKP-1 expression and MAPK activation by PARP-1 in oxidative stress: A new mechanism for the cytoplasmic effect of PARP-1 activation. Free. Radic. Boil. Med..

[13] Gibson B.A., Zhang Y., Jiang H., Hussey K.M., Shrimp J.H., Lin H., Schwede F., Yu Y., Kraus W.L. (2016). Chemical Genetic Discovery of PARP Targets Reveals a Role for PARP-1 in Transcription Elongation. Science.

[14] Marjanović M.P., Crawford K., Ahel I. (2017). PARP, transcription and chromatin modeling. Semin. Cell Dev. Boil..

[15] Kraus W.L. (2008). Transcriptional control by PARP-1: Chromatin modulation, enhancer-binding, coregulation, and insulation. Curr. Opin. Cell Boil..

[16] Krishnakumar R., Kraus W.L. (2010). The PARP side of the nucleus: Molecular actions, physiological outcomes, and clinical targets. Mol. Cell.

[17] Ji Y., Tulin A.V. (2010). The roles of PARP1 in gene control and cell differentiation. Curr. Opin. Genet. Dev..

[18] Kraus W.L. (2015). PARPs and ADP-Ribosylation: 50 Years … and Counting. Mol. Cell.

[19] D’Amours D., Desnoyers S., D’Silva I., Poirier G.G. (1999). Poly(ADP-ribosyl)ation reactions in the regulation of nuclear functions. Biochem. J..

[20] Kronenberg H.M. (2003). Developmental regulation of the growth plate. Nature.

[21] Betts J.G., Desaix P. Bone Tissue and the Skeletal System. https://courses.lumenlearning.com/austincc-ap1/chapter/bone-tissue-and-the-skeletal-system/.

[22] Hall B.K. (1987). Earliest Evidence of Cartilage and Bone Development in Embryonic Life. Clin. Orthop. Relat. Res..

[23] Mbalaviele G., Veis D.J. (2018). Inflammasomes in Bone Diseases. Exp. Suppl..

[24] Mbalaviele G., Novack D.V., Schett G., Teitelbaum S.L. (2017). Inflammatory osteolysis: A conspiracy against bone. J. Clin. Investig..

[25] Novack D.V., Mbalaviele G. (2016). Osteoclasts-Key Players in Skeletal Health and Disease. Microbiol. Spectr..

[26] Pittenger M.F. (1999). Multilineage Potential of Adult Human Mesenchymal Stem Cells. Science.

[27] Peister A., Mellad J.A., Larson B.L., Hall B.M., Gibson L.F., Prockop D.J. (2004). Adult stem cells from bone marrow (MSCs) isolated from different strains of inbred mice vary in surface epitopes, rates of proliferation, and differentiation potential. Blood.

[28] Charbord P., Tavian M., Humeau L., Péault B. (1996). Early ontogeny of the human marrow from long bones: An immunohistochemical study of hematopoiesis and its microenvironment. Blood.

[29] Chen L.T., Weiss L. (1975). The development of vertebral bone marrow of human fetuses. Blood.

[30] Seike M., Omatsu Y., Watanabe H., Kondoh G., Nagasawa T. (2018). Stem cell niche-specific Ebf3 maintains the bone marrow cavity. Genes Dev..

[31] Alippe Y., Wang C., Ricci B., Xiao J., Qu C., Zou W., Novack D.V., Abu-Amer Y., Civitelli R., Mbalaviele G. (2017). Bone matrix components activate the NLRP3 inflammasome and promote osteoclast differentiation. Sci. Rep..

[32] Alippe Y., Mbalaviele G. (2019). Omnipresence of inflammasome activities in inflammatory bone diseases. Semin. Immunopathol..

[33] Charles J.F., Hsu L.-Y., Niemi E.C., Weiss A., Aliprantis A.O., Nakamura M.C. (2012). Inflammatory arthritis increases mouse osteoclast precursors with myeloid suppressor function. J. Clin. Investig..

[34] Jacome-Galarza C.E., Lee S.-K., Lorenzo J.A., Aguila H.L. (2013). Identification, characterization, and isolation of a common progenitor for osteoclasts, macrophages, and dendritic cells from murine bone marrow and periphery. J. Bone Miner. Res..

[35] Jacquin C., Gran D.E., Lee S.K., Lorenzo J.A., Aguila H.L. (2006). Identification of multiple osteoclast precursor populations in murine bone marrow. J. Bone Miner. Res..

[36] Jacome-Galarza C.E., Percin G.I., Muller J.T., Mass E., Lazarov T., Eitler J., Rauner M., Yadav V.K., Crozet L., Bohm M. (2019). Developmental origin, functional maintenance and genetic rescue of osteoclasts. Nature.

[37] Gu R., Santos L.L., Ngo D., Fan H., Singh P.P., Fingerle-Rowson G., Bucala R., Xu J., Quinn J.M.W., Morand E.F. (2015). Macrophage migration inhibitory factor is essential for osteoclastogenic mechanisms in vitro and in vivo mouse model of arthritis. Cytokine.

[38] Romas E., Bakharevski O., Hards D.K., Kartsogiannis V., Quinn J.M.W., Ryan P.F.J., Martin T.J., Gillespie M.T. (2000). Expression of osteoclast differentiation factor at sites of bone erosion in collagen-induced arthritis. Arthritis Rheum..

[39] Kanatani M., Sugimoto T., Takahashi Y., Kaji H., Kitazawa R., Chihara K. (1998). Estrogen via the Estrogen Receptor Blocks cAMP-Mediated Parathyroid Hormone (PTH)-Stimulated Osteoclast Formation. J. Bone Miner. Res..

[40] Dirckx N., Moorer M.C., Clemens T.L., Riddle R.C. (2019). The role of osteoblasts in energy homeostasis. Nat. Rev. Endocrinol..

[41] Lerner U.H., Ohlsson C. (2015). The WNT system: Background and its role in bone. J. Intern. Med..

[42] Sartawi Z., Schipani E., Ryan K.B., Waeber C. (2017). Sphingosine 1-phosphate (S1P) signalling: Role in bone biology and potential therapeutic target for bone repair. Pharmacol. Res..

[43] Meshcheryakova A., Mechtcheriakova D., Pietschmann P. (2017). Sphingosine 1-phosphate signaling in bone remodeling: Multifaceted roles and therapeutic potential. Expert Opin. Ther. Tar..

[44] Pederson L., Ruan M., Westendorf J.J., Khosla S., Oursler M.J. (2008). Regulation of bone formation by osteoclasts involves Wnt/BMP signaling and the chemokine sphingosine-1-phosphate. Proc. Natl. Acad. Sci. USA.

[45] Quint P., Ruan M., Pederson L., Kassem M., Westendorf J.J., Khosla S., Oursler M.J. (2013). Sphingosine 1-Phosphate (S1P) Receptors 1 and 2 Coordinately Induce Mesenchymal Cell Migration through S1P Activation of Complementary Kinase Pathways. J. Boil. Chem..

[46] Park J.H., Lee N.K., Lee S.Y. (2017). Current Understanding of RANK Signaling in Osteoclast Differentiation and Maturation. Mol. Cells.

[47] Sato K., Suematsu A., Nakashima T., Takemoto-Kimura S., Aoki K., Morishita Y., Asahara H., Ohya K., Yamaguchi A., Takai T. (2006). Regulation of osteoclast differentiation and function by the CaMK-CREB pathway. Nat. Med..

[48] Huh J.-E., Lee W.I., Kang J.W., Nam D., Choi D.-Y., Park D.-S., Lee S.H., Lee J.-D. (2014). Formononetin Attenuates Osteoclastogenesis via Suppressing the RANKL-Induced Activation of NF-κB, c-Fos, and Nuclear Factor of Activated T-Cells Cytoplasmic 1 Signaling Pathway. J. Nat. Prod..

[49] Kim J.H., Kim N. (2014). Regulation of NFATc1 in Osteoclast Differentiation. J. Bone Metab..

[50] Yasui T., Tsutsumi S., Aburatani H., Hirose J., Nakamura K., Tanaka S. (2011). Epigenetic regulation of osteoclast differentiation: Possible involvement of Jmjd3 in the histone demethylation of Nfatc1. J. Bone Miner. Res..

[51] Collins P.E., Mitxitorena I., Carmody R.J. (2016). The Ubiquitination of NF-κB Subunits in the Control of Transcription. Cells.

[52] Kim J.H., Kim K., Jin H.M., Song I., Youn B.U., Lee S.H., Choi Y., Kim N. (2010). Negative feedback control of osteoclast formation through ubiquitin-mediated down-regulation of NFATc1. J. Biol. Chem..

[53] Nayak A., Glöckner-Pagel J., Vaeth M., Schumann J.E., Buttmann M., Bopp T., Schmitt E., Serfling E., Berberich-Siebelt F. (2009). Sumoylation of the Transcription Factor NFATc1 Leads to Its Subnuclear Relocalization and Interleukin-2 Repression by Histone Deacetylase. J. Boil. Chem..

[54] Chen N.-M., Neesse A., Dyck M.L., Steuber B., Koenig A.O., Lubeseder-Martellato C., Winter T., Forster T., Bohnenberger H., Kitz J. (2017). Context-Dependent Epigenetic Regulation of Nuclear Factor of Activated T Cells 1 in Pancreatic Plasticity. Gastroenterology.

[55] Yasui T., Hirose J., Aburatani H., Tanaka S. (2011). Epigenetic regulation of osteoclast differentiation. Ann. N. Y. Acad. Sci..

[56] Beranger G.E., Momier D., Rochet N., Quincey D., Guigonis J.M., Samson M., Carle G.F., Scimeca J.C. (2006). RANKL Treatment Releases the Negative Regulation of the Poly(ADP-Ribose) Polymerase-1 on Tcirg1 Gene Expression During Osteoclastogenesis. J. Bone Miner. Res..

[57] Beranger G.E., Momier D., Guigonis J.-M., Samson M., Carle G.F., Scimeca J.-C., Guigonis J., Scimeca J. (2007). Differential Binding of Poly(ADP-Ribose) Polymerase-1 and JunD/Fra2 Accounts for RANKL-Induced Tcirg1 Gene Expression During Osteoclastogenesis. J. Bone Miner. Res..

[58] Beranger G.E., Momier D., Rochet N., Carle G.F., Scimeca J.C. (2008). Poly(adp-ribose) polymerase-1 regulates Tracp gene promoter activity during RANKL-induced osteoclastogenesis. J. Bone Miner. Res..

[59] Chen J., Sun Y., Mao X., Liu Q., Wu H., Chen Y. (2010). RANKL Up-regulates Brain-type Creatine Kinase via Poly(ADP-ribose) Polymerase-1 during Osteoclastogenesis. J. Boil. Chem..

[60] Petrilli V., Herceg Z., Hassa P.O., Patel N.S., Paola R.D., Cortes U., Dugo L., Filipe H.-M., Thiemermann C., Hottiger M.O. (2004). Noncleavable poly(ADP-ribose) polymerase-1 regulates the inflammation response in mice. J. Clin. Investig..

[61] Robaszkiewicz A., Qu C., Wisnik E., Ploszaj T., Mirsaidi A., Kunze F.A., Richards P.J., Cinelli P., Mbalaviele G., Hottiger M.O. (2016). ARTD1 regulates osteoclastogenesis and bone homeostasis by dampening NF-kappaB-dependent transcription of IL-1beta. Sci. Rep..

[62] Wang C., Xu C.-X., Alippe Y., Qu C., Xiao J., Schipani E., Civitelli R., Abu-Amer Y., Mbalaviele G. (2017). Chronic inflammation triggered by the NLRP3 inflammasome in myeloid cells promotes growth plate dysplasia by mesenchymal cells. Sci. Rep..

[63] Wang C., Qu C., Alippe Y., Bonar S.L., Civitelli R., Abu-Amer Y., O Hottiger M., Mbalaviele G. (2016). Poly-ADP-ribosylation-mediated degradation of ARTD1 by the NLRP3 inflammasome is a prerequisite for osteoclast maturation. Cell Death Dis..

[64] Oláh G., Szczesny B., Brunyánszki A., López-García I.A., Gero D., Radak Z., Szabo C. (2015). Differentiation-Associated Downregulation of Poly(ADP-Ribose) Polymerase-1 Expression in Myoblasts Serves to Increase Their Resistance to Oxidative Stress. PLoS ONE.

[65] Castri P., Lee Y.J., Ponzio T., Maric D., Spatz M., Bembry J.H. (2014). Poly(ADP-ribose) polymerase-1 and its cleavage products differentially modulate cellular protection through NF-κB-dependent signaling. BBA-Mol. Cell Res..

[66] Zerfaoui M., Errami Y., Naura A.S., Suzuki Y., Kim H., Ju J., Liu T., Hans C.P., Kim J.G., Elmageed Z.Y.A. (2010). Poly(ADP-ribose) polymerase-1 is a determining factor in Crm1-mediated nuclear export and retention of p65 NF-kappa B upon TLR4 stimulation. J. Immunol..

[67] Valdor R., Schreiber V., Saenz L., Martínez T., Muñoz-Suano A., Domínguez-Villar M., Ramírez P., Parrilla P., Aguado E., Garcia-Cozar F. (2008). Regulation of NFAT by poly(ADP-ribose) polymerase activity in T cells. Mol. Immunol..

[68] Kameoka M., Ota K., Tetsuka T., Tanaka Y., Itaya A., Okamoto T., Yoshihara K. (2000). Evidence for regulation of NF-kappaB by poly(ADP-ribose) polymerase. Biochem. J..

[69] Olabisi O.A., Soto-Nieves N., Nieves E., Yang T.T.C., Yang X., Yu R.Y.L., Suk H.Y., Macian F., Chow C.-W. (2008). Regulation of Transcription Factor NFAT by ADP-Ribosylation. Mol. Cell. Boil..

[70] Malireddi R.K.S., Ippagunta S., Lamkanfi M., Kanneganti T.-D. (2010). Cutting edge: Proteolytic inactivation of poly(ADP-ribose) polymerase 1 by the Nlrp3 and Nlrc4 inflammasomes. J. Immunol..

[71] Qu C., Bonar S.L., Hickman-Brecks C.L., Abu-Amer S., McGeough M.D., Peña C.A., Broderick L., Yang C., Kading J. (2015). NLRP3 mediates osteolysis through inflammation-dependent and -independent mechanisms. FASEB. J..

[72] Erener S., Pétrilli V., Kassner I., Minotti R., Castillo R., Santoro R., Hassa P.O., Tschopp J., Hottiger M.O. (2012). Inflammasome-Activated Caspase 7 Cleaves PARP1 to Enhance the Expression of a Subset of NF-κB Target Genes. Mol. Cell.

[73] García S., Bodaño A., González A., Forteza J., Gómez-Reino J.J., Conde C. (2006). Partial protection against collagen antibody-induced arthritis in PARP-1 deficient mice. Arthritis Res. Ther..

[74] Kunze F.A., Bauer M., Komuczki J., Lanzinger M., Gunasekera K., Hopp A.K., Lehmann M., Becher B., Müller A., Hottiger M.O. (2019). ARTD1 in Myeloid Cells Controls the IL-12/18–IFN-γ Axis in a Model of Sterile Sepsis, Chronic Bacterial Infection, and Cancer. J. Immunol..

[75] Oliver F.J., Murcia J.M., Nacci C., Decker P., Andriantsitohaina R., Muller S., de la Rubia G., Stoclet J.C., de Murcia G. (1999). Resistance to endotoxic shock as a consequence of defective NF-κB activation in poly (ADP-ribose) polymerase-1 deficient mice. EMBO J..

[76] Burkart V., Wang Z.-Q., Radons J., Heller B., Herceg Z., Stingl L., Wagner E.F., Kolb H. (1999). Mice lacking the poly(ADP-ribose) polymerase gene are resistant to pancreatic beta-cell destruction and diabetes development induced by streptozocin. Nat. Med..

[77] Mabley J.G., Jagtap P., Perretti M., Getting S.J., Salzman A.L., Virag L., Szabo E., Soriano F.G., Liaudet L., Abdelkarim G.E. (2001). Anti-inflammatory effects of a novel, potent inhibitor of poly (ADP-ribose) polymerase. Inflamm. Res..

[78] Levaot N., Voytyuk O., Dimitriou I., Sircoulomb F., Chandrakumar A., Deckert M., Krzyzanowski P.M., Scotter A., Gu S., Janmohamed S. (2011). Loss of Tankyrase-mediated destruction of 3BP2 is the underlying pathogenic mechanism of cherubism. Cell.

[79] Ueki Y., Lin C.-Y., Senoo M., Ebihara T., Agata N., Onji M., Saheki Y., Kawai T., Mukherjee P.M., Reichenberger E. (2007). Increased Myeloid Cell Responses to M-CSF and RANKL Cause Bone Loss and Inflammation in SH3BP2 “Cherubism” Mice. Cell.

[80] Levaot N., Simoncic P., Dimitriou I., Scotter A., Rose J.L., Willett T., Ng A., Wang C., Janmohamed S., Grynpas M. (2011). 3BP2 deficient mice are osteoporotic with impaired osteoblast and osteoclast functions. J. Clin. Invest..

[81] Fujita S., Mukai T., Mito T., Kodama S., Nagasu A., Kittaka M., Sone T., Ueki Y., Morita Y. (2018). Pharmacological inhibition of tankyrase induces bone loss in mice by increasing osteoclastogenesis. Bone.

[82] Jiang Q., Paramasivam M., Aressy B., Wu J., Bellani M., Tong W., Seidman M.M., Greenberg R.A. (2015). MERIT40 cooperates with BRCA2 to resolve DNA interstrand cross-links. Genes Dev..

[83] Nagy Z., Kalousi A., Furst A., Koch M., Fischer B., Soutoglou E. (2016). Tankyrases Promote Homologous Recombination and Check Point Activation in Response to DSBs. PLoS Genet..

[84] Zhong L.L., Ding Y., Bandyopadhyay G., Waaler J., Börgeson E., Smith S., Zhang M., Phillips S.A., Mahooti S., Mahata S.K. (2016). The PARsylation activity of tankyrase in adipose tissue modulates systemic glucose metabolism in mice. Diabetologia.

[85] Yeh T.-Y.J., Beiswenger K.K., Li P., Bolin K.E., Lee R.M., Tsao T.-S., Murphy A.N., Hevener A.L., Chi N.-W. (2009). Hypermetabolism, Hyperphagia, and Reduced Adiposity in Tankyrase-Deficient Mice. Diabetes.

[86] Mariotti L., Pollock K., Guettler S. (2017). Regulation of Wnt/beta-catenin signalling by tankyrase-dependent poly(ADP-ribosyl)ation and scaffolding. Br. J. Pharmacol..

[87] Guettler S., LaRose J., Petsalaki E., Gish G., Scotter A., Pawson T., Rottapel R., Sicheri F. (2011). Structural Basis and Sequence Rules for Substrate Recognition by Tankyrase Explain the Basis for Cherubism Disease. Cell.

[88] Ueki Y., Tiziani V., Santanna C., Fukai N., Maulik C., Garfinkle J., Ninomiya C., Doamaral C., Peters H., Habal M. (2001). Mutations in the gene encoding c-Abl-binding protein SH3BP2 cause cherubism. Nat. Genet..

[89] Mukai T., Fujita S., Morita Y. (2019). Tankyrase (PARP5) Inhibition Induces Bone Loss through Accumulation of Its Substrate SH3BP2. Cells.

[90] Yoshitaka T., Mukai T., Kittaka M., Alford L.M., Masrani S., Ishida S., Yamaguchi K., Yamada M., Mizuno N., Olsen B.R. (2014). Enhanced TLR-MYD88 signaling stimulates autoinflammation in SH3BP2 cherubism mice and defines the etiology of cherubism. Cell Rep..

[91] Haigis M.C., Mostoslavsky R., Haigis K.M., Fahie K., Christodoulou D.C., Murphy A.J., Valenzuela D.M., Yancopoulos G.D., Karow M., Blander G. (2006). SIRT4 inhibits glutamate dehydrogenase and opposes the effects of calorie restriction in pancreatic beta cells. Cell.

[92] Moynihan K.A., Grimm A.A., Plueger M.M., Bernal-Mizrachi E., Ford E., Cras-Méneur C., Permutt M.A., Imai S.I. (2005). Increased dosage of mammalian Sir2 in pancreatic beta cells enhances glucose-stimulated insulin secretion in mice. Cell Metab..

[93] Bordone L., Motta M.C., Picard F., Robinson A., Jhala U.S., Apfeld J., McDonagh T., Lemieux M., McBurney M., Szilvasi A. (2006). Sirt1 regulates insulin secretion by repressing UCP2 in pancreatic beta cells. PLoS Biol..

[94] Rodgers J.T., Lerin C., Haas W., Gygi S.P., Spiegelman B.M., Puigserver P. (2005). Nutrient control of glucose homeostasis through a complex of PGC-1alpha and SIRT1. Nature.

[95] Canto C., Auwerx J. (2012). Targeting sirtuin 1 to improve metabolism: All you need is NAD(+)?. Pharmacol. Rev..

[96] Mao Z., Hine C., Tian X., Van Meter M., Au M., Vaidya A., Seluanov A., Gorbunova V., Hine C. (2011). SIRT6 promotes DNA repair under stress by activating PARP1. Science.

[97] Rezazadeh S., Yang D., Tombline G., Simon M., Regan S.P., Seluanov A., Gorbunova V. (2019). SIRT6 promotes transcription of a subset of NRF2 targets by mono-ADP-ribosylating BAF170. Nucleic Acids Res..

[98] Van Meter M., Kashyap M., Rezazadeh S., Geneva A.J., Morello T.D., Seluanov A., Gorbunova V. (2014). SIRT6 represses LINE1 retrotransposons by ribosylating KAP1 but this repression fails with stress and age. Nat. Commun..

[99] Liszt G., Ford E., Kurtev M., Guarente L. (2005). Mouse Sir2 Homolog SIRT6 Is a Nuclear ADP-ribosyltransferase. J. Boil. Chem..

[100] North B.J., Marshall B.L., Borra M.T., Denu J.M., Verdin E. (2003). The human Sir2 ortholog, SIRT2, is an NAD+-dependent tubulin deacetylase. Mol. Cell.

[101] Bhardwaj A., Das S. (2016). SIRT6 deacetylates PKM2 to suppress its nuclear localization and oncogenic functions. Proc. Natl. Acad. Sci. USA.

[102] Pan P.W., Feldman J.L., Devries M.K., Dong A., Edwards A.M., Denu J.M. (2011). Structure and Biochemical Functions of SIRT6. J. Boil. Chem..

[103] Dominy J.E., Lee Y., Jedrychowski M.P., Chim H., Jurczak M.J., Camporez J.P., Ruan H.-B., Feldman J., Pierce K., Mostoslavsky R. (2012). The deacetylase Sirt6 activates the acetyltransferase GCN5 and suppresses hepatic gluconeogenesis. Mol. Cell.

[104] Kawahara T.L., Michishita E., Adler A.S., Damian M., Berber E., Lin M., Mccord R.A., Ongaigui K.C., Boxer L.D., Chang H.Y. (2009). SIRT6 links histone H3 lysine 9 deacetylation to NF-kappaB-dependent gene expression and organismal life span. Cell.

[105] Abu-Amer Y. (2013). NF-κB signaling and bone resorption. Osteoporos. Int..

[106] Lee H.-S., Ka S.-O., Lee S.-M., Lee S.-I., Park J.-W., Park B.-H. (2013). Overexpression of Sirtuin 6 Suppresses Inflammatory Responses and Bone Destruction in Mice With Collagen-Induced Arthritis. Arthritis Rheum..

[107] Mostoslavsky R., Chua K.F., Lombard D.B., Pang W.W., Fischer M.R., Gellon L., Liu P., Mostoslavsky G., Franco S., Murphy M.M. (2006). Genomic Instability and Aging-like Phenotype in the Absence of Mammalian SIRT6. Cell.

[108] Zhang D., Jing J., Lou F., Li R., Ping Y., Yu F., Wu F., Yang X., Xu R., Li F. (2018). Evidence for excessive osteoclast activation in SIRT6 null mice. Sci. Rep..

[109] Zhang D.-M., Cui D.-X., Xu R.-S., Zhou Y.-C., Zheng L.-W., Liu P., Zhou X.-D. (2016). Phenotypic research on senile osteoporosis caused by SIRT6 deficiency. Int. J. Oral Sci..

[110] Sugatani T., Agapova O., Malluche H.H., Hruska K.A. (2015). SIRT6 deficiency culminates in low-turnover osteopenia. Bone.

[111] Moon Y.J., Zhang Z., Bang I.H., Kwon O.K., Yoon S.-J., Kim J.R., Lee S., Bae E.J., Park B.-H. (2019). Sirtuin 6 in preosteoclasts suppresses age- and estrogen deficiency-related bone loss by stabilizing estrogen receptor α. Cell Death Differ..

[112] Park S.J., Huh J.-E., Shin J., Park D.R., Ko R., Jin G.-R., Seo D.-H., Kim H.-S., Shin H.-I., Oh G.T. (2016). Sirt6 cooperates with Blimp1 to positively regulate osteoclast differentiation. Sci. Rep..

[113] Long F. (2011). Building strong bones: Molecular regulation of the osteoblast lineage. Nat. Rev. Mol. Cell Boil..

[114] Gibon E., Lu L., Goodman S.B. (2016). Aging, inflammation, stem cells, and bone healing. Stem Cell Res. Ther..

[115] Abdelmagid S.M., Barbe M.F., Safadi F.F. (2015). Role of inflammation in the aging bones. Life Sci..

[116] Lo M.L., Pannone G., Santarelli A., Lo R.L., De L.A., Rubini C., Bambini F., Bufo P., Dioguardi M., Procaccini M. (2014). Expression of poly(ADP-ribose) polymerase in bone regeneration. J. Biol. Regul. Homeost. Agents..

[117] Chow W.Y., Rajan R., Muller K.H., Reid D.G., Skepper J.N., Wong W.C., Brooks R.A., Green M., Bihan D., Farndale R.W. (2014). NMR Spectroscopy of Native and in Vitro Tissues Implicates PolyADP Ribose in Biomineralization. Science.

[118] Hegedűs C., Robaszkiewicz A., Lakatos P., Szabo E., Virág L. (2015). Poly(ADP-ribose) in the bone: From oxidative stress signal to structural element. Free. Radic. Boil. Med..

[119] Robaszkiewicz A., Erdélyi K., Kovács K., Kovács I., Bai P., Rajnavölgyi É., Virág L. (2012). Hydrogen peroxide-induced poly(ADP-ribosyl)ation regulates osteogenic differentiation-associated cell death. Free. Radic. Boil. Med..

[120] Robaszkiewicz A., Valkó Z., Kovács K., Hegedűs C., Bakondi E., Bai P., Virág L. (2014). The role of p38 signaling and poly(ADP-ribosyl)ation-induced metabolic collapse in the osteogenic differentiation-coupled cell death pathway. Free. Radic. Boil. Med..

[121] Zhang S.Z., Lu Z.F., Xu Y.J., Shi H.F., Liu Y.Z., Rui Y.J. (2018). STEEL participates in fracture healing through upregulating angiogenesis-related genes by recruiting PARP 1. Eur. Rev. Med. Pharmacol. Sci..

[122] Kishi Y., Fujihara H., Kawaguchi K., Yamada H., Nakayama R., Yamamoto N., Fujihara Y., Hamada Y., Satomura K., Masutani M. (2015). PARP Inhibitor PJ34 Suppresses Osteogenic Differentiation in Mouse Mesenchymal Stem Cells by Modulating BMP-2 Signaling Pathway. Int. J. Mol. Sci..

[123] Majewski P.M., Thurston R.D., Ramalingam R., Kiela P.R., Ghishan F.K. (2010). Cooperative role of NF-κB and poly(ADP-ribose) polymerase 1 (PARP-1) in the TNF-induced inhibition of PHEX expression in osteoblasts. J. Biol. Chem..

[124] Majewski P.M., Kędzierska U., Banasiak Ł., Kiela P. (2018). Significance of NF-κB signaling and PARP1 activity in the TNF-induced inhibition of PHEX gene expression in human osteoblasts. Acta Biochim. Pol..

[125] Huang S.-M.A., Mishina Y.M., Liu S., Cheung A., Stegmeier F., Michaud G.A., Charlat O., Wiellette E., Zhang Y., Wiessner S. (2009). Tankyrase inhibition stabilizes axin and antagonizes Wnt signalling. Nature.

[126] Matsumoto Y., Rose J.L., Kent O.A., Wagner M.J., Narimatsu M., Levy A.D., Omar M.H., Tong J., Krieger J.R., Riggs E. (2016). Reciprocal stabilization of ABL and TAZ regulates osteoblastogenesis through transcription factor RUNX2. J. Clin. Investig..

[127] Piao J., Tsuji K., Ochi H., Iwata M., Koga D., Okawa A., Morita S., Takeda S., Asou Y. (2013). Sirt6 regulates postnatal growth plate differentiation and proliferation via Ihh signaling. Sci. Rep..

[128] Zhang P., Liu Y., Wang Y., Zhang M., Lv L., Zhang X., Zhou Y. (2017). SIRT6 promotes osteogenic differentiation of mesenchymal stem cells through BMP signaling. Sci. Rep..

[129] Sun H., Wu Y., Fu D., Liu Y., Huang C. (2014). SIRT6 Regulates Osteogenic Differentiation of Rat Bone Marrow Mesenchymal Stem Cells Partially via Suppressing the Nuclear Factor-κB Signaling Pathway. Stem Cells.

[130] Bredella M.A., Torriani M., Ghomi R.H., Thomas B.J., Brick D.J., Gerweck A.V., Rosen C.J., Klibanski A., Miller K.K. (2011). Vertebral bone marrow fat is positively associated with visceral fat and inversely associated with IGF-1 in obese women. Obesity (Silver Spring).

[131] Bredella M.A., Torriani M., Ghomi R.H., Thomas B.J., Brick D.J., Gerweck A.V., Harrington L.M., Breggia A., Rosen C.J., Millerb K.K. (2011). Determinants of bone mineral density in obese premenopausal women. Bone.

[132] Hawkes C.P., Mostoufi-Moab S. (2019). Fat-bone interaction within the bone marrow milieu: Impact on hematopoiesis and systemic energy metabolism. Bone.

[133] Smink J.J., Leutz A. (2012). Instruction of mesenchymal cell fate by the transcription factor C/EBPβ. Gene.

[134] Pekala P.H., Lane M.D., Watkins P.A., Moss J. (1981). On the mechanism of preadipocyte differentiation. Masking of poly(ADP-ribose) synthetase activity during differentiation of 3T3-L1 preadipocytes. J. Boil. Chem..

[135] Erener S., Mirsaidi A., Hesse M., Tiaden A.N., Ellingsgaard H., Kostadinova R., Donath M.Y., Richards P.J., Hottiger M. (2012). ARTD1 deletion causes increased hepatic lipid accumulation in mice fed a high-fat diet and impairs adipocyte function and differentiation. FASEB J..

[136] Erener S., Hesse M., Kostadinova R., Hottiger M.O. (2012). Poly(ADP-ribose)polymerase-1 (PARP1) controls adipogenic gene expression and adipocyte function. Mol. Endocrinol..

[137] Bai P., Cantó C., Oudart H., Brunyánszki A., Cen Y., Thomas C., Yamamoto H., Huber A., Kiss B., Houtkooper R.H. (2011). PARP-1 inhibition increases mitochondrial metabolism through SIRT1 activation. Cell Metab..

[138] Nagy L., Rauch B., Balla N., Ujlaki G., Kis G., Abdul-Rahman O., Kristóf E., Sipos A., Antal M., Tóth A. (2019). Olaparib induces browning of in vitro cultures of human primary white adipocytes. Biochem. Pharmacol..

[139] Luo X., Ryu K.W., Kim D.S., Nandu T., Medina C.J., Gupte R., Gibson B.A., Soccio R.E., Yu Y.H., Gupta R.K. (2017). PARP-1 Controls the Adipogenic Transcriptional Program. by PARylating C/EBPbeta and Modulating Its Transcriptional Activity. Mol. Cell.

[140] Mirza M.R., Monk B.J., Herrstedt J., Oza A.M., Mahner S., Redondo A., Fabbro M., Ledermann J.A., Lorusso D., Vergote I. (2016). Niraparib Maintenance Therapy in Platinum-Sensitive, Recurrent Ovarian Cancer. New Engl. J. Med..

[141] Marchetti C., Imperiale L., Gasparri M.L., Palaia I., Pignata S., Boni T., Bellati F., Panici P.B. (2012). Olaparib, PARP1 inhibitor in ovarian cancer. Expert Opin. Investig. Drugs.

[142] Keung M.Y.T., Wu Y., Vadgama J.V. (2019). PARP Inhibitors as a Therapeutic Agent for Homologous Recombination Deficiency in Breast Cancers. J. Clin. Med..

[143] Tutt A., Robson M., Garber J.E., Domchek S.M., Audeh M.W., Weitzel J.N., Friedlander M., Arun B., Loman N., Schmutzler R.K. (2010). Oral poly(ADP-ribose) polymerase inhibitor olaparib in patients with BRCA1 or BRCA2 mutations and advanced breast cancer: a proof-of-concept trial. Lancet.

[144] Mizutani A., Yashiroda Y., Muramatsu Y., Yoshida H., Chikada T., Tsumura T., Okue M., Shirai F., Fukami T., Yoshida M. (2018). RK-287107, a potent and specific tankyrase inhibitor, blocks colorectal cancer cell growth in a preclinical model. Cancer Sci..

[145] Paulsen J.E., Pedersen N.M., Kries J.P.V., Waaler J., Machon O., Tumova L., Dinh H., Korinek V., Wilson S.R., Eide T.J. (2012). A Novel Tankyrase Inhibitor Decreases Canonical Wnt Signaling in Colon Carcinoma Cells and Reduces Tumor Growth in Conditional APC Mutant Mice. Cancer Res..

[146] Li C., Zheng X., Han Y., Lv Y., Lan F., Zhao J. (2018). XAV939 inhibits the proliferation and migration of lung adenocarcinoma A549 cells through the WNT pathway. Oncol. Lett..

[147] Cheng H., Li X., Wang C., Chen Y., Li S., Tan J., Tan B., He Y. (2019). Inhibition of tankyrase by a novel small molecule significantly attenuates prostate cancer cell proliferation. Cancer Lett..

[148] Jia J., Qiao Y., Pilo M.G., Cigliano A., Liu X., Shao Z., Calvisi D.F., Chen X. (2017). Tankyrase inhibitors suppress hepatocellular carcinoma cell growth via modulating the Hippo cascade. PLoS ONE.

[149] Stratford E.W., Daffinrud J., Munthe E., Castro R., Waaler J., Krauss S., Myklebost O. (2014). The tankyrase-specific inhibitor JW74 affects cell cycle progression and induces apoptosis and differentiation in osteosarcoma cell lines. Cancer Med..

[150] Martins-Neves S.R., Paiva-Oliveira D.I., Fontes-Ribeiro C., Bovée J.V., Cleton-Jansen A.-M., Gomes C.F. (2018). IWR-1, a tankyrase inhibitor, attenuates Wnt/β-catenin signaling in cancer stem-like cells and inhibits in vivo the growth of a subcutaneous human osteosarcoma xenograft. Cancer Lett..

[151] Gröschel S., Hübschmann D., Raimondi F., Horak P., Warsow G., Fröhlich M., Klink B., Gieldon L., Hutter B., Kleinhenz K. (2019). Defective homologous recombination DNA repair as therapeutic target in advanced chordoma. Nat. Commun..

